# Burnt Sugarcane Harvesting – Cardiovascular Effects on a Group of Healthy Workers, Brazil

**DOI:** 10.1371/journal.pone.0046142

**Published:** 2012-09-27

**Authors:** Cristiane Maria Galvão Barbosa, Mário Terra-Filho, André Luis Pereira de Albuquerque, Dante Di Giorgi, Cesar Grupi, Carlos Eduardo Negrão, Maria Urbana Pinto Brandão Rondon, Daniel Godoy Martinez, Tânia Marcourakis, Fabiana Almeida dos Santos, Alfésio Luís Ferreira Braga, Dirce Maria Trevisan Zanetta, Ubiratan de Paula Santos

**Affiliations:** 1 Pulmonary Division - Heart Institute(InCor), Hospital das Clínicas da Faculdade de Medicina da Universidade de São Paulo, São Paulo, São Paulo, Brazil; 2 FUNDACENTRO, São Paulo, São Paulo, Brazil; 3 Hypertension Unit, Heart Institute(InCor), Hospital das Clínicas da Faculdade de Medicina da Universidade de São Paulo, São Paulo, São Paulo, Brazil; 4 Electrocardiology Unit, Heart Institute(InCor), Hospital das Clínicas da Faculdade de Medicina da Universidade de São Paulo, São Paulo, São Paulo, Brazil; 5 Unit of Cardiovascular Rehabilitation and Exercise Physiology, Heart Institute (InCor), Hospital das Clínicas da Faculdade de Medicina da Universidade de São Paulo, São Paulo, São Paulo, Brazil; 6 Department of Clinical and Toxicological Analyses, University of São Paulo Pharmacological Sciences School, São Paulo, Brazil; 7 Environmental Epidemiology Study Group, Laboratory of Experimental Air Pollution, Department of Pathology, Faculdade de Medicina da Universidade de São Paulo, São Paulo, São Paulo, Brazil; 8 Environmental Exposure and Risk Assessment Group, Catholic University of Santos, Santos, São Paulo, Brazil; 9 Department of Epidemiology, University of São Paulo School Public Health, São Paulo, Brazil; California Pacific Medicial Center Research Institute, United States of America

## Abstract

**Background:**

Brazil is the world's largest producer of sugarcane. Harvest is predominantly manual, exposing workers to health risks: intense physical exertion, heat, pollutants from sugarcane burning.

**Design:**

Panel study to evaluate the effects of burnt sugarcane harvesting on blood markers and on cardiovascular system.

**Methods:**

Twenty-eight healthy male workers, living in the countryside of Brazil were submitted to blood markers, blood pressure, heart rate variability, cardiopulmonary exercise testing, sympathetic nerve activity evaluation and forearm blood flow measures (venous occlusion plethysmography) during burnt sugarcane harvesting and four months later while they performed other activities in sugar cane culture.

**Results:**

Mean participant age was 31±6.3 years, and had worked for 9.8±8.4 years on sugarcane work. Work during the harvest period was associated with higher serum levels of Creatine Kinase – 136.5 U/L (IQR: 108.5–216.0) vs. 104.5 U/L (IQR: 77.5–170.5), (p = 0.001); plasma Malondialdehyde–7.5±1.4 µM/dl vs. 6.9±1.0 µM/dl, (p = 0.058); Glutathione Peroxidase – 55.1±11.8 Ug/Hb vs. 39.5±9.5 Ug/Hb, (p<0.001); Glutathione Transferase– 3.4±1.3 Ug/Hb vs. 3.0±1.3 Ug/Hb, (p = 0.001); and 24-hour systolic blood pressure – 120.1±10.3 mmHg vs. 117.0±10.0 mmHg, (p = 0.034). In cardiopulmonary exercise testing, rest-to-peak diastolic blood pressure increased by 11.12 mmHg and 5.13 mmHg in the harvest and non-harvest period, respectively. A 10 miliseconds reduction in rMSSD and a 10 burst/min increase in sympathetic nerve activity were associated to 2.2 and 1.8 mmHg rises in systolic arterial pressure, respectively.

**Conclusion:**

Work in burnt sugarcane harvesting was associated with changes in blood markers and higher blood pressure, which may be related to autonomic imbalance.

## Introduction

Brazil is the world's largest producer of sugar and ethanol from sugarcane, with 570 million tons in 2007/2008 harvest [1]. Although industrial harvesting processes utilize technological methods, manual harvesting is still the predominant method of harvesting sugarcane, and it employs nearly 500,000 workers throughout the country. This is a seasonal activity. For seven months per year, to receive an average monthly wage of US$ 700.00 a sugar cane worker must cut approximately 10 tons of sugarcane daily, in journeys of eight hour and twenty minutes, six days a week, under high temperatures in the fields, due to the climate and the heat from burning sugarcane, and receiving inappropriate reposition of water and electrolytes. Moreover, they are exposed to pollutants released during the cutting of burnt sugarcane.

Exposure to air pollution is associated with increased cardio-respiratory morbimortality, and most studies on the subject are related to urban pollution (industrial/vehicular origin) [2], [3], [4], [5]. The studies on outdoor air pollution caused by biomass burning have focused more on the respiratory effects [6], [7] than on the cardiovascular effects [8], [9]. Study carried out with sugar cane workers found in their urine levels of 1-hydroxipirene, an exposure markers for polycyclic aromatic hydrocarbons, 10 times higher during the harvest season [10].

There are currently no published studies on the cardiovascular risks associated with manual harvesting of sugarcane, which combines physical and thermal overload as well as exposure to pollutants under conditions that are present in countries such as Brazil, India, Philippines, Latin and Central America countries. It has been reported sugar cane workers' diseases and sudden deaths in the last decade [11], [12].

Our objective was to evaluate the occurrence of cardiovascular effects and the possible mechanisms involved in these events associated with the harvesting of burnt sugarcane.

## Methods

### Study population and period

This is an observational study with repeated measures conducted on 28workers at a sugar and ethanol mill. All participants were Caucasian, male, healthy, between 18 and 50 years of age and had no clinical history or use of medications for cardiopulmonary disease.

The study participants were evaluated at two periods: at the end of burnt sugarcane harvest (October–November 2007) and at the end of period when burnt sugarcane was not being harvested (March–April 2008), when the cutters performed cleanup and planting of unburned sugarcane. As they did not earn by productivity in this period, activity was physically less intense. These time points are henceforth referred to as the harvest and non-harvest periods, respectively.

The Research Ethics Committee of the University of São Paulo Medical School approved the study and all participants signed consent forms.

### Examination procedures

**Figure 1:**
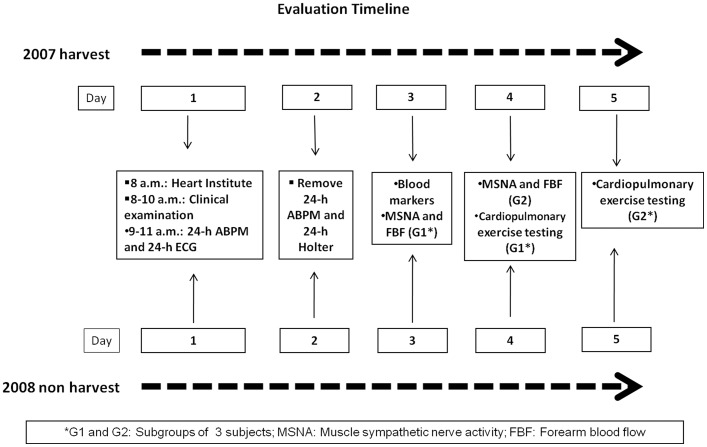
Flow Chart - Evaluation sequence: five groups of six participants.

Because there are many risk factors as physical and thermal overload as well as exposure to air pollutants, we decide to use a wide range of effect indicators. These markers have been used in exercise, environmental, and occupational health studies. During both periods, workers were divided into groups of five or six. After working all week, they were brought from the countryside to the Heart Institute in São Paulo city, where they underwent several examinations over five consecutive days. Evaluations were conducted sequentially to avoid any changes between exams (Figure 1).

1. The participants answered a questionnaire that was developed for the study and involved the following: data regarding the work during both periods, work time, prior occupational exposures, smoking, and the presence of general and respiratory symptoms. Anthropometric measurements were taken at this moment.
2. Blood markers: fibrinogen, thrombin time (TT), prothrombin time (PT), platelet count, creatine kinase (CK), lactate dehydrogenase (LDH), lipid profile, serum calcium, serum sodium and C-reactive protein (CRP) by high sensitive immunology assay (Dade Behring Marburg GmbH, Germany), erythrocyte glutathione peroxidase (GPx), Glutathione-S-Transferase (GST) and glutathione reductase (GR) measured by spectrophotometry [13], [14] and plasma malondialdehyde (MDA) measured by high-performance liquid chromatography [15].
3. Twenty-four-hour ambulatory blood pressure monitoring (ABPM-24 hour) was performed using a Spacelabs-90207 monitor (Spacelabs Medical Inc., USA). Measurements were taken every 10-minutes during the day (5a.m. to 10 p.m.) and every 20-minutes at night (10 p.m. to 5 a.m.), according to standards that have been defined and used with our services [16].
4. Twenty-four-hour electrocardiography monitoring (ECG-24 hour): was performed according to a method that was previously used in another study by our group [17]. Records were analyzed to obtain heart rate variability (HRV) indicators, including the standard deviation of normal RR intervals (SDNN), the standard deviation of sequential five-minute RR interval means (SDANN), and the root mean square of differences between NN adjacent intervals (rMSSD).
5. Cardiopulmonary exercise testing (CPET): a ramp symptom-limited CPET was performed on a cycle (Corival, The Netherlands), consisting in a 2-min period of rest, a 2-min period of warm-up (unloaded pedaling) followed by an incremental work-rate period (increase of 20 W/min) [18]. Oxygen saturation (SpO_2_) by pulse oximetry (NONIN-ONYX, model 9500, Plymouth, MN, USA) and electrocardiography (Welch Allyn CardioPerfect, Inc, NY) were monitored continuously. The following variables were recorded breath-by-breath (CardiO_2_ System, MGC): oxygen consumption (VO_2_), minute-ventilation (V_E_), carbon dioxide output (VCO_2_), tidal volume (V_T_), respiratory rate, respiratory exchange rate (RER) and heart rate (HR).
6. Muscle sympathetic nerve activity (MSNA) was directly measured from the peroneal nerve using the technique of microneurography, as described elsewhere [19]. Multiunit postganglionic muscle sympathetic nerve recordings were made using a tungsten microelectrode (tip-diameter 5–15 µm). Muscle sympathetic bursts were identified by visual inspection and were expressed as burst per 100 heartbeats.
7. Forearm blood flow (FBF) was measured by venous occlusion plethysmography (Hokanson, Bellevue, WA, USA), according to methods that have been described elsewhere [19]. FBF (mL/min/100 mL of tissue) was determined based on a minimum of four separate readings. Forearm vascular conductance (units) was calculated by dividing the forearm blood flow by the mean blood pressure (oscillometrically measured).

### Work and environment assessment

**Figure 2:**
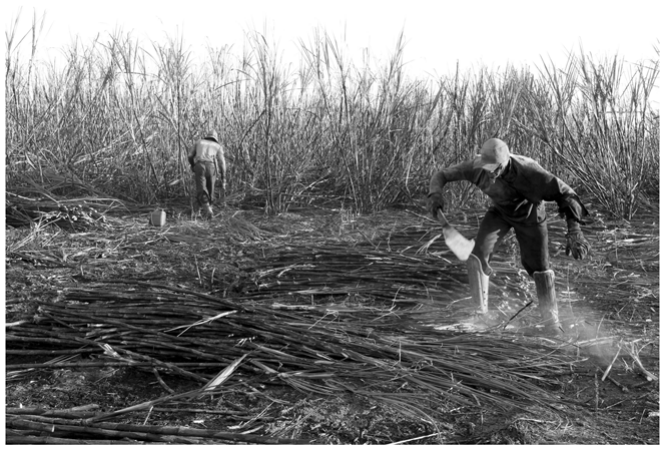
Worker cutting burnt sugarcane.

During both periods, we recorded the concentration of Particulate Matter (PM) with a diameter of 2.5 (PM_2.5_) using a DustTrak Aerosol Monitor, model-8520 (TSI-Inc., MN, USA) that was calibrated and adjusted prior to the measurements with a flow rate of 1.7 l/min [20]. On the same days and locations, the temperature (°C) and relative air humidity (%) were measured using a digital thermohygrometer (Dataloger-TFA, 3030.15, Germany). To record environmental variables, an activity cycle was sought to best represent the typical activities of the workers (Figure 2).

During the harvest period, activities began approximately 4–6 hours after the end of burning. PM and climate variables were measured in the sugarcane field at three 6-hour periods for three consecutive days during the harvest period and during the cutting and weeding of non-burnt sugarcane in the non-harvest period, when there is exposure to resuspended soil particulate matter.

To estimate the level of heat exposure to which the workers were subjected, we obtained the Wet Bulb Globe Temperature (WBGT) Index, a heat exposure indicator [21]. We used a Thermal Stress Meter, model-500 by Quest. The equipment was placed near the worker and readings were made during one workday, according to ACGIH recommendations [22].

### Statistical analysis

Categorical variables are presented as absolute numbers and percentages, whereas continuous variables are given as the mean±standard deviation (SD) or as the median and the interquartile range (IQR). Descriptive analysis were performed for the study variables and the results obtained during the harvest and non-harvest periods were compared by statistical tests for repeated measurements (paired t-test, Wilcoxon rank-test or McNemar's test, as appropriate). Differences of PM_2.5_ and climate variables measurements, between harvest and non-harvest periods, were tested using Mann-Whitney U test.

When the p-value of the differences in measurements for both periods evaluated was≤0.10, linear regression analysis was carried out using a generalized estimating equation (GEE) with robust standard error estimators to evaluate the effects of harvesting work. Adjustments were made for age, body mass index (BMI) and smoking (nonsmoking as a reference), assuming equal correlation of the measurements for each subject (exchangeable correlation). Socioeconomic status was quite similar among the participants and was not included in the analysis. The effects of HRV and MSNA on blood pressure (BP) were evaluated controlling for harvest, age, BMI and smoking (***BP∼HRV or MSNA+harvesting+age+smoking+BMI***). The GEE function was obtained from StatLib (http://lib.stat.cmu.edu/) and the analysis was performed on S-Plus® 8.0 for Windows Statistics software, Insightful Corp., Seattle, WA.

## Results

### Particulate matter, climate and heat exposure

Table 1 shows data for PM_2,5_ concentrations, temperature and relative air humidity (RAH) at the sugarcane fields during the two periods. The PM_2.5_ concentration was higher during the harvest period. There was no significant difference in temperature, and RAH was significantly higher during the non-harvest period. WBGT varied between 18.1 and 28.4°C, with the highest value observed between 11a.m.and 12p.m.

**Table 1:** Descriptive analyses of fine particles (PM_2.5_), ambient temperature and relative humidity during harvest and non-harvest periods at the sugarcane field.

Variables	Harvest	Non-harvest	p-value^a^
	Median (IQR^b^)	Median (IQR)	
**PM_2.5_** (µg/m^3^)	87.0(70.0–100,0)	50.0(40.5–61.5)	<0.001
**Ambient Temperature** (°C)	28.1(25.6–33.0)	28.2(25.6–31.8)	0.500
**Relative Humidity** (%)	49.0(40.0–59.0)	65.0(61.0–72.8)	<0.001

a : Mann-Whitney test;

b : interquartile range.

### Individual evaluations

The mean worker age was 31±6.3 years (range: 21–45 years), and the average length of time spent employed in sugarcane harvesting was 9.8±8.4 years. Nineteen workers (68%) were never-smokers and nine (32%) were light smokers (7±4.2 pack-years). All workers had less than eight years of schooling, earned between 500 and 800 US dollars per month, according to the amount of cut cane. The housing conditions were similar, living on the outskirts of small towns in the region. None had the automotive vehicle itself.

As the similar cultural habits and socioeconomic conditions, these workers have the same food habits. They carry to work on the field the meal prepared the day before and stored in bowls partially saving thermal food temperature. However, the fact that there is loss of heat by the time of the meal gave this group the nickname of “*cold-meal workers*”. Usually they take rice and beans, potatoes and some animal protein in the form of egg, meat, or chicken

History of previous chronic disease was absent in all participants and none presented infectious or traumatic events when evaluated.

Table 2 presents the general data for the participants in both periods. The workers showed significantly lower body weight during the harvest period as well as smaller abdominal circumference and BMI. The serum HDL presented higher levels during harvest when compared to non-harvest period. On the other hand, diastolic and, in a lesser extent, the systolic blood pressure were higher during harvest period.

**Table 2:** General Characteristics of participants during harvest and non-harvest periods in 28 participants.

Variables	Harvest	Non-harvest	p-value
**BMI** a[kg/m^2^ (mean ± SD^b^)]	22.6±2.7	23.4±2.9	<0.001^c^
**Weight** [kg (median; IQR^d^)]	64.5 (61.0–69.5)	67.0 (2.0–73.5)	<0.001^e^
**Abdcirc** f[cm (median; IQR)]	80.0 (75.0–84.0)	83.0 (78.0–87.5)	0.002^e^
**Total Cholesterol** [mg/dL(mean± SD)]	*159.9±38.1*	*151.2±33.5*	*0.041* c
**HDL** g **Cholesterol** [mg/dL(mean± SD)]	*50.0±10.7*	*42.5±8.2*	*<0.00* c
**LDL** h **Cholesterol** [mg/dL(mean± SD)]	*90.0±26.1*	*88.8±27.0*	*0.689* c
**Triglycerides** [mg/dL(mean± SD)]	*102.6±64.2*	*106.7±91.4*	*0.663* c
**Heart rate** [BPM^i^(mean ± SD]	60.8±8.8	58.7±9.1	0.237^c^
**SBP** j[mmHg (mean ± SD)]	125.36±14,81	118.07±17.8	0.046^c^
**DBP** k[mmHg (mean ± SD)]	78.00±12.96	70.57±13.88	0.013^c^

a : body mass index;

b : standard deviation;

c : paired t test;

d : interquartile range;

e : Wilcoxon rank test;

f : abdominal circumference;

g **: High Density Lipoprotein;**

h **: Low Density Lipoprotein;**

i **: beats per minute;**

j **: systolic blood pressure;**

k **: diastolic blood pressure.**

Sixteen workers (51.7%) reported nasal itching and rhinorrhea during the harvest period, versus four (14.3%) in the non-harvest period (p<0.01); ten workers (35.7%) mentioned cramping during the harvest period, compared with three (10.7%) in the non-harvest period (p = 0.02); seven workers (25.0%) reported a dry cough during the harvest period, versus only one (3.6%) in the non-harvest period (p = 0.02).

During the harvest period, the workers reported a daily water intake of 5–10 liters. The average weight of the sugarcane that was cut daily, as reported by the workers, was 11 tons (range: 7–14), differently from non-harvest period, when workers receive a pre-defined payment by month and, therefore, the job is less intensive.

**Figure 3:**
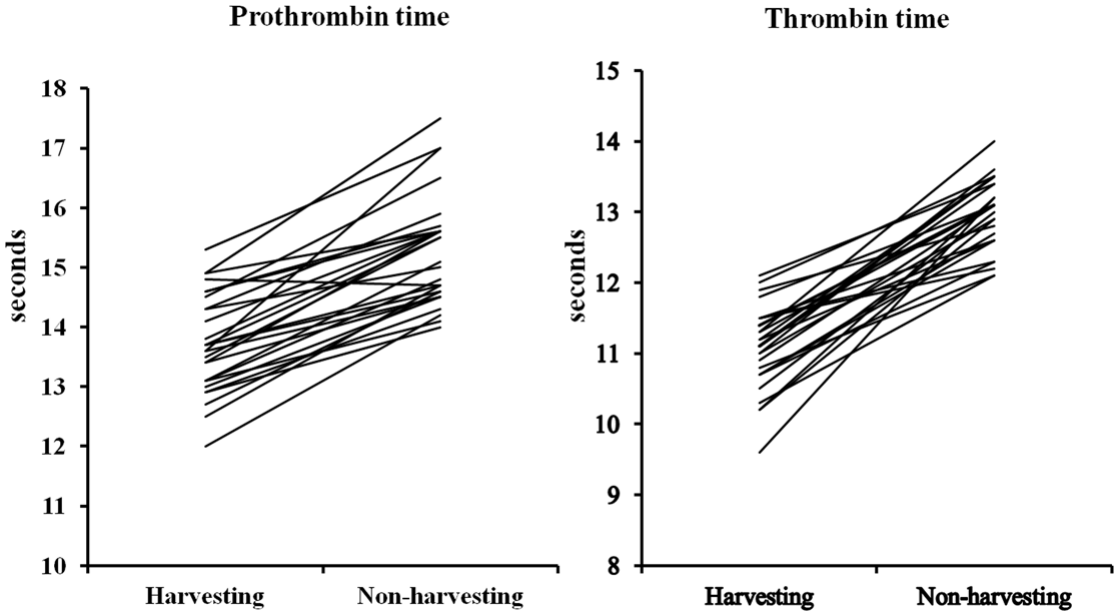
Prothrombin and Thrombin time in harvest and non-harvest periods (n:28).

Table 3 and Figure 3 shows the blood marker results for the workers during both periods. The CK and LDH levels were higher, while CRP, calcium, TT and PT were significantly lower during the harvest period. Sixteen (57%) workers showed sodium levels below 140 meq/L during the harvest period, versus six (21.5%) in the non-harvest period. Fibrinogen levels were higher in the harvest period, but the differences were not significant.

**Table 3:** Descriptive analyses of blood markers during harvest and non-harvest periods and the estimated effects of harvest using regressions analysis for repeated measures in 28 participants.

Variables	Periods		Univariate Analysis	Multiple Analysis^a^	
	Harvest	Non-harvest	p-value	RC (95%CI)^b^	p-value
**Sodium** (mEq/L)	139.6±2.3^c^	141.6±1.6^c^	0.046^d^	−0.86 (−1.84; 0.12)	0.085
**Calcium** (mg/dl)	9.1±0.4^c^	9.3±0.3^c^	0.013^d^	−0.21(−0.36; −0.07)	0.004
**CK** e(U/L)	136.5 (108.5–216.0)^f^	104.5 (77.5–170.5)^f^	<0.001^g^	39.06 (19.24; 58.87)	<0.001
**LDH** h(U/L)	156.6±22.0^c^	148.1±23.9^c^	0.028^d^	9.44 (2.10; 16,78)	0.012
**CRP** i(mg/L)	2.5 (1.6–5.3)^f^	4.4 (1.7–15.2)^f^	<0.001^g^	−4.46 (−6,89; −2.02)	<0.001
**PT** j(s)	13.7±0.8^c^	15.3±0.9^c^	<0.001^d^	−1.50 (−1.77; −1.23)	<0.001
**TT** k(s)	11.1±0.6^c^	13.0±0.5^c^	<0.001^d^	−1.86 (−2.10; −1.62)	<0.001

a : multiple analysis adjusted for age, body mass index and smoking;

b : RegressionCoefficientand95%Confidence interval;

c : mean and standard deviation;

d : paired t test;

e : creatine kinase;

f : median and interquartile range;

g : Wilcoxon runk test;

h : lactate dehydrogenase;

i : C-Reactive Protein;

j : prothrombin time;

k : thrombin time.

The enzymes GR, GST and GPX activity, as well as MDA levels, were higher in the harvest period and the difference was significant for the GST and GPX (Table 4).

**Table 4:** Glutathione transferase (GST), glutathione peroxidase (GPX), glutathione reductase (GR), and malondialdehyde (MDA) during harvest and non-harvest periods in 28 participants.

Variables	Periods		Paired t test	Multiple analysis^a^	
	Harvest (mean±SD^b^)	Non-harvest (mean±SD)	p-value	RC (95%CI)^c^	p-value
**GST** d(Ug/Hb)	3.38±1.27	3.01±1.31	0.019	0.39(0.16; 0.63)	0.01
**GPX** e (Ug/Hb)	55.06±11.84	39.48±9.45	0.001	15.31(9.81; 20.82)	<0.001
**GR** f (Ug/Hb)	3.05±0.97	2.91±1.13	0.401	0.23(−0.08; 0.54)	0.143
**MDA** g (µM/dL)	7.50±1.42	6.89±1.01	0.088	6.4(−0.26; 13.06)	0.057

a : multiple analysis adjusted for age, body mass index and smoking;

b : standard deviation;

c : regression coefficient and 95% confidence interval;

d : Glutathione - S- Transferase;

e : Glutathione Peroxidase;

f : Glutathione Reductase;

g : Malondialdehyde.

### Blood pressure and heart rate variability

The 24 hours Systolic and Mean BP were significantly higher during the harvest period (Table 5). HRV indicators tended to be higher in the harvest period, although the difference in multiple regression analysis was significant only for SDANN, the estimate of long-term components of HRV. The frequency domain variables did not present statistically significant variations.

**Table 5:** Ambulatory Blood Pressure Monitoring (ABPM) and Heart Rate Variability (HRV) during harvest and non-harvest periods in 28 participants.

Variables	Periods (mean±SD^a^)		Paired t test	Multiple analysis^b^	
	Harvest	Non-harvest	p-value	RC (95%CI)^c^	p-value
**24 h ABPM** d					
**SBP** e (mmHg)	120.1±10.3	117.0±10.0	0.110	3.69 (0.27; 7.11)	0.034
**MBP** f(mmHg)	86.8±9.4	84.4±8.6	0.103	2.95 (0.15; 5.75)	0.039
**24 h ECG-HRV** g					
**SDNN** h **(ms** i **)**	187.1±37.7	178.6±40.5	0.030	5.81 (−2.77; 14.39)	0.184
**SDANN** j **(ms)**	161.6±30.4	149.0±34.0	0.006	11.22 (2.40; 20.03)	0.013
**RMSSD** k **(ms)**	47.5±19.1	47.6±19.4	0.957	−2.52 (−7.19;2.15)	0,290

a : standard deviation;

b : multiple analysis adjusted for age, body mass index and smoking;

c : regression coefficient and 95% confidence interval;

d : Twenty-four-hour Ambulatory blood pressure monitoring;

e : Systolic blood pressure;

f : Mean blood pressure;

g : Twenty-four-hour electrocardiogram-heart rate variability;

h : standard deviation of normal RR intervals;

i : milliseconds;

j : Standard Deviation of Sequential Five-Minute R-R Interval Means;

k : root mean square of successive differences in adjacent NN intervals.

### Sympathetic nerve and vascular measures

Among the evaluated individuals, 25 adequately completed these tests during both stages. The values for MSNA and FBF at rest and during handgrip exercise tended to be higher during the harvest period, although the differences were not significant (Table 6).

**Table 6:** Descriptive analyses of muscle sympathetic nerve activity (MSNA) and forearm blood flow (FBF) at rest and during handgrip exercise in 25 participants.

Variable	Harvest mean±SD^a^	Non-harvest mean±SD	p-value^b^
**MSNA (bursts/100HB** c **)** d			
**Baseline**	38.9±11.0	36.1±8.6	0.056
**1′ exercise**	38.2±10.9	36.0±8.9	0.339
**2′ exercise**	40.9±9.3	39.0±8.9	0.440
**3′ exercise**	45.5±10.1	43.5±10.4	0.460
**FBF (ml/min/100 ml of tissue)**			
**Baseline**	2.9±1.4	2.8±1.2	0.657
**1′ exercise**	3.3±1.5	3.1±1.4	0.559
**2′ exercise**	3.3±1.8	3.0±1.3	0.356
**3′ exercise**	3.3±1.9	3.2±1.4	0.602

a : standard deviation;

b : p-value: Paired T-test;

c : heart beats;

d : during 30% of maximum voluntary contraction.

### Cardiopulmonary exercise testing

**Figure 4:**
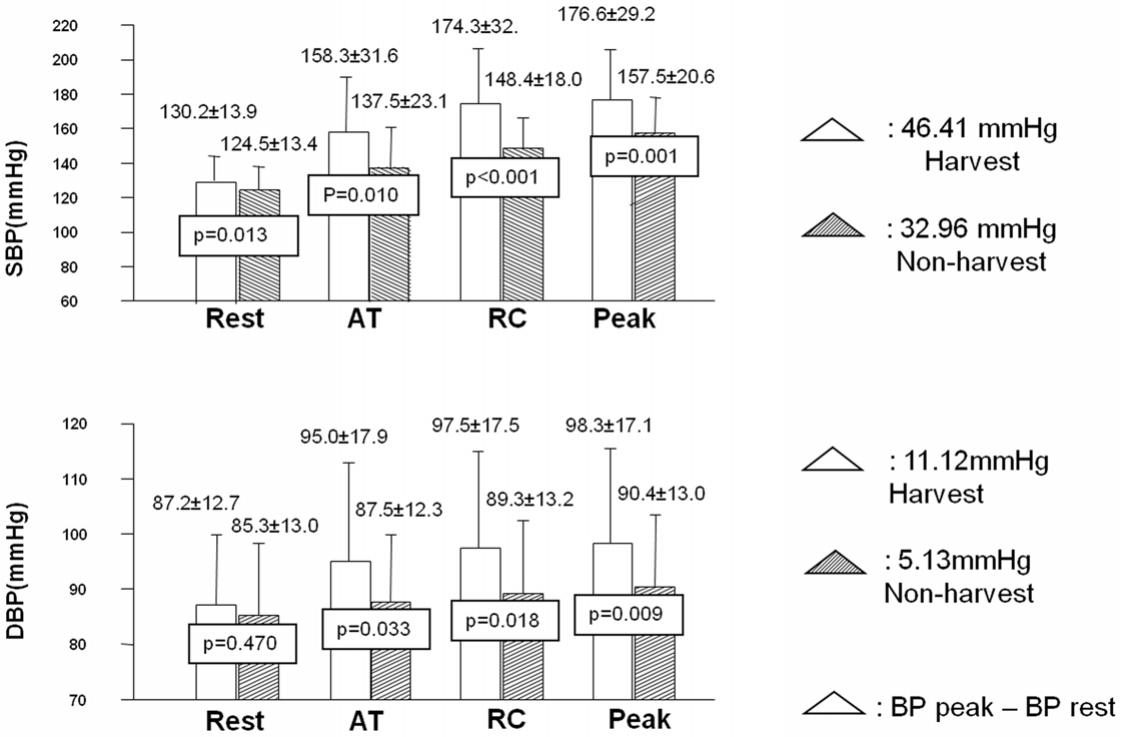
Systolic (SBP) and diastolic (DBP) blood pressure variation during cardiopulmonary exercise testing in each period, mean±SD, (n:24). AT: Anaerobic Threshold; RC: respiratory compensation; Δ = differences between peak and rest in mmHg.

Among the evaluated individuals, 24 adequately completed this test during both stages. The mean duration of exercise was 12 minutes. The peak O_2_ uptake (peak VO_2_), the O_2_ pulse and the peak systolic/diastolic BP were significantly higher during the harvest period (Table 7). Figure 4 shows that the systolic (SBP) and diastolic (DBP) blood pressures were higher in the harvest period during all test stages. DBP showed an 11.1 mmHg increase at the end of exercise during the harvest period (p<0.001), versus 5.1 mmHg in the non-harvest period (p = 0.064).

**Table 7:** Univariate analysis and the estimated effects of harvest using regression models for repeated measures on cardiopulmonary exercise testing in 24 participants.

Variables	Periods		Paired t test	Multipleanalysis^a^	
	Harvest^b^	Non-harvest^b^	p-value	RC (95%CI)^c^	p-value
**Peak VO_2_** (ml/Kg/min)	40.4±6.9	36.5±6,3	0.005	2.86 (0.68; 5.05)	0.010
**Peak O_2_** (ml/beat)	16.3±3.0	14.9±3.2	0.009	1.51 (0.64; 2.38)	0.001
**SBP** d **rest**(mmHg)	130.2±13.9	124.5±13.5	0.013	6,94 (3.02; 10.86)	0.001
**SBPpeak**(mmHg)	176.6±29.9	157.6±20.6	0.001	21.59 (11.58; 31.60)	<0.001
**DBP** e **rest**(mmHg)	87.2±12.7	85.3±13.0	0.470	3.69 (−1.12;8.50)	0.133
**DBP peak** (mmHg)	98.3±17.1	90.4±13.0	0.009	10.15 (4.98; 15.32)	<0.001

a : multiple analysis adjusted for age, body mass index and smoking;

b : mean± standard deviation;

c : regression coefficient and 95% confidence interval;

d : systolic blood pressure;

e : diastolic blood pressure.

Multiple analyses showed that the BP values recorded by ABPM during the harvest period were significantly associated with reductions in HRV and increases in MSNA (Table 8). A 10 ms reduction in SDNN and in rMSSD and a 10 burst/min increase in sympathetic nerve activity were associated with 0.7, 2.2, and 1.8 mmHg rises in 24-hours SBP, respectively.

**Table 8:** Effects on blood pressure associated with heart rate variability (HRV) and muscle sympathetic nerve activity (10×).

Variable	24 h SBP^a^		24 h DBP^b^		24 h MBP^c^	
	Regression Coefficients (95%CI^d^)	p value	Regression Coefficients (95%CI)	p value	Regression Coefficients (95%CI)	p value
**HRV** e **(n:28)**						
*SDNN* f *(ms)*	−0.7 (−1.5; 0.03)	0.058	−0.62 (−1.2; −0.1)	0.029	−0.7 (−1.3; −0.1)	0.02
*RMSSD* g *(ms)*	−2.2 (−4.1; −0.4)	0.019	−1.6 (−3.0; −2.5)	0.021	−2.4 (−3.9; −0.8)	0.009
*HF* h *(ms)*	−5.6 (−9.4; −1.8)	0.004	−3.7 (−6.8; −0.6)	0.021	−4.8 (−8.1; −1.4)	0.005
*LF* i *(ms)*	−3.7 (−7.7; 0.2)	0.065	−3.0 (−5.9; −0.06)	0.046	−3.5 (−6.8; −0.1)	0.044
*LF/HF*	28.3 (17.3; 39.3)	<0.001	−7.4 (−26.4; 11.5)	0,519	26.4 (18.8; 33.9)	<0.001
**MSNA** j **/MVC** k **(n = 25)**	1.8 (0.1; 3.5)	0.035	1.4 (0.05; 2.7)	0.049	1.6 (0.8; 2.4)	0.041

Multiple analysis adjusted for age, BMI and smoking;

a : systolic blood pressure;

b : diastolic blood pressure;

c : men blood pressure;

d : confidence interva;

e : heart rate variability;

f : standard deviation of normal RR intervals;

g : root mean square of differences between NN adjacent intervals;

h : high frequence;

i : low frequence;

j : Muscle sympathetic- nerve activity-bursts/100 Heart beats;

k : maximum voluntary contraction at 30%.

## Discussion

This study revealed that sugarcane workers are subjected to several risks, such as physical overload in hot conditions and exposure to pollutants. Combined, these conditions, which were observed during the harvest period, are related to changes in cardiovascular and blood markers.

In agreement with other authors [11], the present findings indicated the adverse conditions to which sugarcane harvesters are subjected. Studies of the movements of workers during sugarcane harvesting have estimated that cutters bend their backs approximately 4,000 times and make close to 3,800 machete strikes during an 8-hour work day [23].

There are currently few published studies evaluating sugarcane workers [10], [20]. The existing studies have evaluated pollutants in the area where the sugarcane is burned and in nearby cities [24]. It is also likely that the PM associated with sugarcane burning presents distinct characteristics during the different periods, as can be observed by the blackish color of the workers' clothes in the harvest period. During burning, (Figure 2) we observed suspended matter present in the sugar cane and on the surface of the top layer of soil, resulting from the burning of waste straw that is released by machete strikes, whereas during the non-burning period the suspended PM contains mainly particles suspended from soil.

The WBTG value was high, reaching 28.43°C, similar to the findings of another study on sugarcane harvesting [23] which that registered 27.9°C. These values surpass the recommended limits for continuous labor [21], suggesting that heat stress can occur over the working months, which demonstrates the need for breaks during the workday. Several studies have related the effect of variations of temperature in the cardiovascular system [24], a condition experienced by workers on a daily basis. In this study, the WBTG varied from 18°C to 28°C, what could explain the reports of hospitalization and deaths that have been described in the sugar cane plantation [20].

Higher levels of CK and LDH are compatible with labor under conditions of physical overload and hydro-electrolyte imbalance, as evidenced in several studies [25], [26]. LDH and CK are biomarkers of muscular damage and may increase during situations of intense exercise, in which cell membranes become more permeable and enzymes are released into the interstitial matrix and reabsorbed via the lymphatic system to enter the bloodstream [26].The observation that CK remained high, even in blood samples measured 60 hours after exercise suggests a state of persistent hyperCKemia, much like that which occurs in athletes [26]. Performing exhaustive exercise in an excessively warm environment increases the risk of muscle lesion [26].

Although serum sodium is a less sensitive marker, the lower sodium and calcium levels observed during the harvest period can arise due to possible hydro-electrolyte disturbances associated with intense sweating and the intake of large quantities of water, which could explain the higher frequency of reported cramping in this period [25], [27]. Another study [28] reported the occurrence of hyponatremia among military personnel during intensive training, due to excessive water intake.

The changes observed in TT and PT may be related to exposure to pollutants, as suggested in other studies [29] and likely indicate increased blood viscosity. A study [30] involving 38 individuals showed that metals present in the water-soluble fraction of air pollution particles have an important role in decreasing the whole-blood coagulation time. These findings [30] and those of a recently published study [31] analyzing the composition and effects of PM arising from vehicular emissions and sugarcane burning found a higher concentration of metals (Ni, Fe, Zn, Mn) in the latter, supporting the findings of short PT and TT observed in our study.

The lower CRP values during the harvest period do not concur with the findings of several studies on pollution [32], [33]. However, other studies did not show an association between exposure to pollutants and CRP [34], [35]. Our findings may be due to the positive effect of increased physical activity during the harvest period. Studies performed on athletes have shown a reduction in CRP after intensive physical training [36], [37]. The same was observed in the lipid profile. The only statistically significant variation was observed for HDL cholesterol, which increased during the harvest period, probably due to the more intense physical activity performed during this period.

The increased activities of antioxidant enzymes during the harvest period may be associated with a greater stimulus by oxidant radicals produced by exercise [38], [39] and inhalation of pollutants [40], [41]. The increased levels of MDA, a lipid peroxidation marker [39], during harvest (Table 4), although not statistically significant (p = 0.057), may suggest cell damage even with increases of GST and GPX enzymes, indicating an upper regulation of antioxidant defense mechanism [42]. This suggests that both processes may be concomitant. Poorly planned physical activity that is excessive in intensity and pace and is associated with exhaustion – similar to what occurs during the harvest period – may also induce the development of oxidative stress [38], [39].

The reduction of SDANN observed in this study, which has matched the ultralow frequency variability, suggests negative influence on cardiac autonomic balance, a known cardiovascular risk factor [43], it has been associated with air pollution exposures [44], [45], [46].

Higher O_2_ pulse and peak VO_2_ levels observed in CPET during the harvest period may be associated with an improvement in the physical conditioning of workers during that period [18]. Peak VO_2_ is an index used to measure performance in athletes from whom high VO_2_max values are expected. One study on sugarcane harvesters in Colombia showed a VO_2_max of 42 ml/kg/min, which is close to the value found in our study [47].The values found in this study were similar to those found in sedentary individuals [48] but lower than those in endurance athletes and higher than those in strength athletes [49]. This results are compatible with the activities of sugar cane workers whose labor requires a combination of physical strength and endurance to cut around tens tons per day of sugar cane and carry them from the field to the trucks.

Blood pressure levels raised more during the harvest period, both at rest and during exercise, which may be associated with several mechanisms. Experimental studies in animals [50] and humans [35] as well as epidemiological studies [17], [51], [52] have shown an association between exposure to vehicular and biomass air pollutants and both acute and chronic elevated BP [53]. The main hypothesis about the effect of air pollution involves oxidative stress and pulmonary inflammation [40] inducing systemic inflammation that could cause vascular dysfunction, endothelial dysfunction, vasoconstriction and cardiac remodeling [50], [54]. An imbalance of the autonomic nervous system (ANS) can occur due to the stimulation of intrapulmonary receptors of the ANS. This imbalance can induce changes in the cardiovascular system, resulting in adrenergic vasoconstriction, increased cardiac output and activation of the renin-angiotensin system, leading to endothelial dysfunction and vasoconstriction [54].

By revealing an increase in ABPM-measured blood pressure, an abnormal DBP response during exercise, and the presence of lipid peroxidation days after the end of exposure, our data suggest a subacute effect associated with systemic stress and inflammation [54]. These effects could account for the ANS imbalance evidenced by the relationship between reduced HRV and increased MSNA and higher BP (Table8), as suggested in a recent study [35].

Although the effects of pollution on the sodium balance in the kidneys are unknown, a possible hydro-electrolyte imbalance resulting from strenuous labor under thermal overload conditions may be an additional factor acting on the renin-angiotensin system and thus contributing to higher BP [55] possibly leading to synergism between PM exposure and high temperatures, as suggested in some studies [56], [57]. Another possible explanation could be a chronic rise in vasopressin levels resulting from the rhythm of water intake after intense sweating – in other words, thirst, which happens daily during work. This increase may be associated with higher BP, as suggested in other studies [58].

The persistently inadequate response of DBP during exercise, even during the non-harvest period, suggests the presence of subacute and chronic changes in the cardiovascular system.

This investigation was delineated to evaluate the possible causes associated to sudden deaths that are observed among sugar cane workers. Besides strenuous outdoor work under unsatisfactory feeding and hydration conditions, they are exposed to particulate matter that could increase adverse effects. Different from other study [59] that showed a small systemic proinflammatory response induced only by intermittent moderate-intense exercise, we observed effects on blood pressure and coagulation markers inversely related with regular physical exercise [60]. Increases of blood pressure (mean, systolic, and diastolic, the last during exercise test) and shortening of thrombin and prothrombin times during harvest period could be attributed to pollution exposure.

In summary, it was not possible to evaluate the isolated impact of each risk factor (air pollution, heat and exertion) on the alterations found (changes in coagulation, oxidative stress and blood pressure). However, these alterations suggest an important impact of the work environment on health. As such, these changes can account for the reports of morbimortality among these workers, which affects the most susceptible and/or those who put forth the most effort to achieve a higher income.

The great number of agricultural workers – as well as other workers, such as miners – facing similar conditions around the world makes this problem a public health issue that deserves more attention from different public administration sectors.

### Limitations

There are limitations in our study. One result from our inability to better describe individual worker exposure during a longer environmental evaluation period. However, the great homogeneity of the work involved in cutting sugarcane and the minor changes in meteorological variables suggest that the variation in PM_2.5_is not relevant, considering that our analyses compared the effects of harvest and non-harvest periods. The existence of a control group could have helped the comparison of certain outcomes, but the challenges in creating a group to compare several conditions (temperature, strenuous exercise, pollution and socioeconomic condition) could not be overcome. The fact that we used repeated measurements to compare the effects of different conditions on the same subjects minimizes this issue.

### Conclusions

Sugarcane work during the harvesting period exposes workers to higher levels of particulate matter, thermal overload, and intense physical exertion, inducing muscle lesion, changes in blood coagulation and in heart rate variability, systemic oxidative stress, and high blood pressure. The autonomic imbalance seems to be one of the mechanisms involved in blood pressure changes.
